# Associated tears of the lateral meniscus in anterior cruciate ligament injuries: risk factors for different tear patterns

**DOI:** 10.1186/s13018-015-0184-x

**Published:** 2015-03-18

**Authors:** Matthias J Feucht, Sebastian Bigdon, Gerrit Bode, Gian M Salzmann, David Dovi-Akue, Norbert P Südkamp, Philipp Niemeyer

**Affiliations:** Department of Orthopedic Surgery and Traumatology, Freiburg University Hospital, Albert Ludwigs University of Freiburg, Hugstetter Straße 55, 79106 Freiburg, Germany

**Keywords:** Anterior cruciate ligament, ACL, Meniscus, Root tear, Contact injury

## Abstract

**Background:**

The pattern of lateral meniscus tears observed in anterior cruciate ligament (ACL)-injured subjects varies greatly and determines subsequent management. Certain tear patterns with major biomechanical consequences should be repaired in a timely manner. Knowledge about risk factors for such tears may help to identify patients in the early posttraumatic phase and subsequently may improve clinical results.

**Methods:**

A database of 268 patients undergoing primary ACL reconstruction was used to identify all patients with isolated ACL tears and patients with an associated tear of the lateral meniscus. Patients who underwent surgery >6 months after the injury were excluded. Based on the arthroscopic appearance of the lateral meniscus, patients were assorted to one of three groups: ‘no tear,’ ‘minor tear,’ and ‘major tear.’ Tear patterns defined as major included root tears, complete radial tears, and unstable longitudinal tears including bucket-handle tears. Univariate analysis was performed by comparing the three groups with regard to gender, age, height, weight, BMI, type of injury (high-impact sport, low-impact sport, and not sports related), and mechanism of injury (non-contact vs. contact). Multivariate logistic regression was carried out to identify independent risk factors for minor and major meniscal tears and to calculate odds ratios (OR).

**Results:**

Two hundred fifteen patients met the inclusion and exclusion criteria. Of those, 56% had isolated ACL tears, 27% had associated minor tears, and 17% had associated major tears of the lateral meniscus. Univariate analysis revealed significant differences between the three groups for gender (*p* = 0.002), age groups (*p* = 0.026), and mechanism of injury (*p* < 0.001). A contact injury mechanism was a risk factor for minor tears (OR: 4.28) and major tears (OR: 18.49). Additional risk factors for major tears were male gender (OR: 7.38) and age <30 years (OR: 5.85).

**Conclusion:**

Male patients, patients <30 years, and particularly patients who sustained a contact injury have a high risk for an associated major lateral meniscus tear. Special attention is therefore necessary in those patients and early referral to magnetic resonance imaging and/or arthroscopy is recommended to allow meniscus repair in a timely manner.

## Background

Meniscus tears are commonly observed in patients with anterior cruciate ligament (ACL) injuries, with a reported prevalence of approximately 55% to 65% [1-6]. Several studies have shown that associated meniscal tears are strong predictors for the development and progression of knee osteoarthritis (OA) as well as worse patient reported outcomes after ACL reconstruction, especially if a partial or total meniscectomy is performed [7-11]. This observation has led to efforts to preserve as much meniscal tissue as possible, and meniscus repair combined with ACL reconstruction is increasingly preferred over meniscectomy [12,13].

Whereas medial meniscus tears are more common in patients with chronic ACL insufficiency, lateral meniscus tears are predominately found in acute ACL injuries [14,15]. Since the complexity of meniscus tears increase in the chronic stage, and tears are less amenable to repair as time passes [16,17], particularly lateral meniscus tears identified in the early posttraumatic phase may be best suitable for repair. The importance of lateral meniscus repair is emphasized by the fact that lateral meniscectomy is associated with a higher risk for osteoarthritis compared to medial meniscectomy [7,9].

The pattern of lateral meniscus tears observed in ACL-injured subjects varies greatly and determines subsequent management. Certain tear patterns, such as incomplete longitudinal tears or complete stable longitudinal tears have only minor consequences on knee joint health and can be left in situ [18-20]. In contrast, other tear patterns, such as root tears, complete radial tears, and bucket-handle tears, are associated with major biomechanical consequences and should be repaired in a timely manner to prevent rapid joint degeneration [21-24].

Different tear patterns of the lateral meniscus in ACL-injured subjects may be associated with different demographic and historical risk factors such as gender, age, body weight, and injury mechanism. Knowledge about such risk factors may help physicians to identify patients with major meniscal tears in the early posttraumatic phase. However, no study so far has analyzed risk factors for different tear patterns of the lateral meniscus in acute and subacute ACL-injured subjects. The purpose of this study was therefore to refine current knowledge about risk factor for associated meniscus tears in ACL-injured subjects by specifically analyzing risk factors for different tear patterns of the lateral meniscus.

## Methods

### Study design

A retrospective cohort design was used to examine the association between different tear patterns of the lateral meniscus in acute and subacute ACL-injured subjects and potential demographic and historical risk factors.

The study protocol was approved by the institutional review board of the University of Freiburg (Project No.: 170/14), and the study was performed in accordance with the Declaration of Helsinki. Informed consent of the patients was not necessary since all data were obtained retrospectively from patient records.

A chart review was performed using our electronic medical record system to identify all patients undergoing primary ACL reconstruction between January 2011 and December 2013. For the purpose of this study, only patients with isolated ACL tears and patients with concomitant tears of the lateral meniscus were included for further analysis. Patients were excluded from the present study if they fulfilled one of the following criteria: concomitant insufficiency of the posterior cruciate ligament, concomitant grade 2 or 3 injury of the medial/lateral collateral ligament, concomitant tear of the medial meniscus, and a history of previous surgery at the index knee. Patient selection was performed after reviewing the preoperative clinical notes and the operation reports, which included documentation of the findings of the knee examination at the time of surgery with the patient under anesthesia and the findings of the diagnostic arthroscopy prior to ligament reconstruction. Since increased time from injury to surgery may contribute to subsequent meniscal tears, we additionally excluded patients who underwent surgery more than six months after the injury.

**Figure 1:**
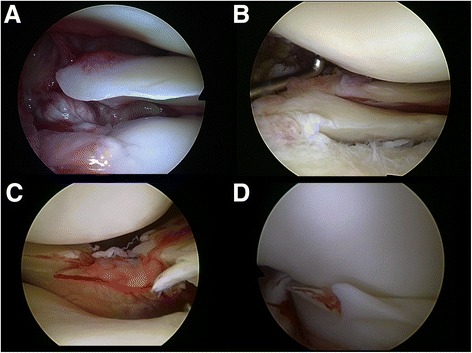
**Lateral meniscus tears defined as major tears.** Root tears (defined as avulsion of the meniscus root **(A)** or complete radial/oblique radial tears within one centimeter from the bony insertion of the lateral meniscus **(B)**); radial split tears **(C)** and unstable longitudinal tears including bucket-handle tears **(D)**.

For medicolegal reasons, standardized photographic documentation of every diagnostic arthroscopy and of crucial steps of each arthroscopic procedure is mandatory at our institution, and all photographs are archived in a picture archiving and communication system (PACS). Therefore, digitalized arthroscopic photographs from the index procedure were available for all patients, which were reviewed by a single observer with extensive experience in arthroscopic knee surgery. Based on the arthroscopic appearance of the lateral meniscus, patients were assorted to one of three groups: ‘no tear,’ ‘minor tear,’ and ‘major tear.’ Tear patterns defined as minor were incomplete longitudinal tears or complete stable longitudinal tears not extending further than 1 cm in front of the popliteus tendon and radial or flap tears involving less than 75% of the meniscal width [18-20,23]. Tear patterns defined as major included root tears (defined as avulsion of the meniscus root or complete radial tears within 1 cm from the bony insertion of the lateral meniscus), complete radial tears with transection of the meniscus (‘radial split tears’), and unstable longitudinal tears including bucket-handle tears (Figure 1) [21-24].

### Data collection

The preoperative clinical notes of all patients were reviewed to collect demographic and historical data. The following variables were considered for the present study: gender, age at surgery, height, weight, body mass index (BMI), type of injury, and mechanism of injury. Patient age was further analyzed by dividing the cohort into two age groups: <30 years and >30 years. For BMI analysis, patients were divided into three groups based on the classification of the World Health Organization [25]: <24.9 kg/m^2^ (normal), 25–29.9 kg/m^2^ (overweight), and >30 kg/m^2^ (obese). The type of injury was defined as the circumstance in which the injury occurred and was classified as high-impact sports-related, low-impact sports-related, and not sports-related injuries. The mechanism of injury was classified as non-contact mechanism or contact mechanism according to the definition of the Hunt Valley II Meeting in 2005 [26]. A non-contact mechanism was assumed if the forces applied to the knee joint resulted from the patients’ own movements and did not involve contact with another person or object. A contact mechanism was assumed if an external force was directly applied to the knee joint or if an external force was applied to the patient but not directly to the injured knee.

### Statistical analysis

Statistical analysis was performed using SPSS software version 21.0 (IBM-SPSS, New York, USA). The level of significance was set at *P* < .05. Continuous variables were calculated as mean ± standard deviation and 95% confidence interval (CI). Categorical variables were reported as count and percentages.

Univariate analysis was performed by comparing the three study groups with regard to gender, age, age groups (<30 years and >30 years), height, weight, BMI, BMI groups (<24.9, 25–29.9, and >30), type of injury (high-impact sports, low-impact sports, and not sports related), and mechanism of injury (non-contact and contact). Normal distribution of continuous variables was evaluated with the Kolmogorov-Smirnov test. Normally distributed continuous variables were compared using one-way analysis of variance (ANOVA) and the *post hoc* Tukey’s honest significant difference test. Non-normal distributed continuous variables and categorical variables were compared using the Kruskal-Wallis test and chi-squared test. *Post hoc* analyses were performed by multiple comparisons using the Mann–Whitney *U* test or Fisher’s exact test. For *post hoc* comparisons, the level of significance was corrected with a Bonferroni adjustment.

Multivariate logistic regression was carried out to identify independent risk factors for minor and major tears. All variables were initially included in the multivariate models, and elimination of non-significant factors was performed using a stepwise backward elimination approach. Level of significance, odds ratios (OR), and 95% CIs were calculated for each variable.

## Results

A total of 268 patients underwent primary ACL reconstruction during the study period. Of these, 215 patients met the inclusion and exclusion criteria and were included in the present study. Thirty-six percent of the included patients were females and 64% were males. Mean age was 27.8 ± 10 years, mean height was 174.8 ± 9.1 cm, mean weight was 75.4 ± 15.2 kg, and mean BMI was 24.6 ± 4.7 kg/m^2^. Sixty-seven percent of the patients were aged <30 years and the BMI was normal in 65%. Most patients (67%) injured their ACL during high-impact sports and a non-contact mechanism was found in 79%.

**Figure 2:**
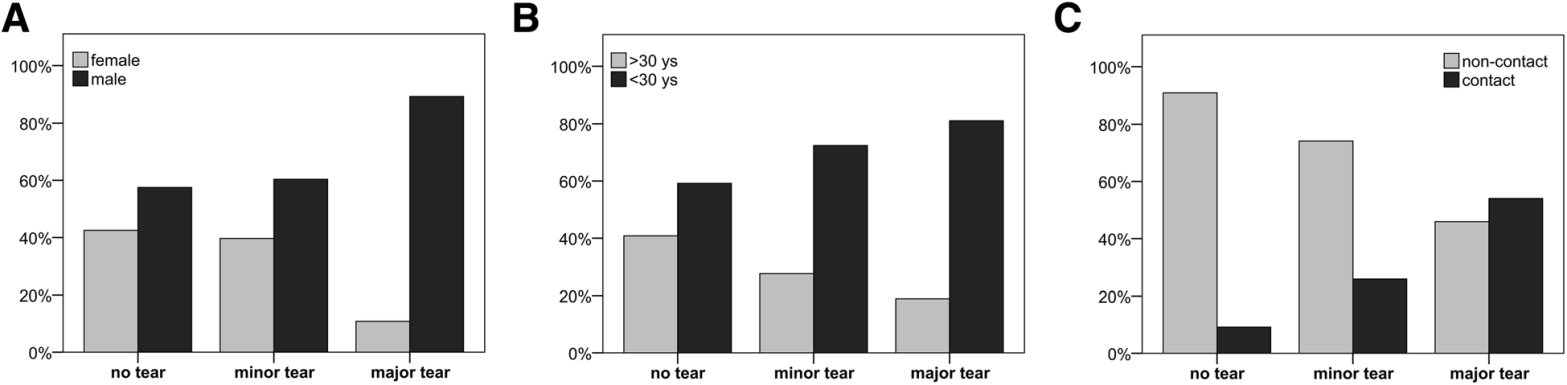
**Significant results.** Distribution of gender **(A)**, age groups **(B)**, and injury mechanism **(C)** within each group. The sole independent risk factor for a minor tear was a contact mechanism. Independent risk factors for major tears were male gender, age <30 years, and a contact mechanism.

Of the 215 included patients, 120 (56%) patients had isolated ACL tears, 58 (27%) had an associated minor lateral meniscus tear, and 37 (17%) had an associated major lateral meniscus tear. The detailed distribution of meniscus tear patterns is shown in Table 1. Patient characteristics of each group and the results of the univariate group comparison are presented in Table 2. Statistically significant differences between the ‘no tear’ and ‘minor tear’ groups were found for the mechanism of injury, with a higher proportion of contact injuries in the minor tear group (*p* = 0.006). Compared to patients with no tear, a significantly higher proportion of male patients (*p* < 0.001), patients <30 years (*p* = 0.015), and contact injuries (*p* < 0.001) were found in patients with major tears. In addition, contact injuries were significantly more common in patients with major tears as compared to patients with minor tears (*p* = 0.009). The results of the multivariate logistic regression analysis are shown in Table 3. The sole independent risk factor for a minor tear was a contact mechanism with an OR of 4.28 (95% CI, 1.74–10.56). Independent risk factors for major tears were male gender (OR, 7.38; 95% CI, 1.97–27.61), age <30 years (OR, 5.85; 95% CI, 1.71–19.94), and a contact mechanism (OR, 18.49; 95% CI, 5.96–57.37) (Figure 2).

**Table 1:** **Distribution of meniscus tear patterns**

**Group**	**Number (%)**
No tear	120 (56)
Minor tear	58 (27)
Incomplete/complete stable longitudinal tear extending <1 cm in front of the popliteus tendon	45 (21)
Radial or flap tear involving <75% of the meniscal width	13 (6)
Major tear	37 (17)
Root tear	22 (10)
Radial split tear	8 (4)
Unstable longitudinal/bucket-handle tear	7 (3)

Percentage values were calculated for the total study population.

**Table 2:** **Univariate analysis** ^a^

**Variable**	**Group**			
	**‘No tear’**	**‘Minor tear’**	**‘Major tear’**	***P*** **value**
Gender				0.002^b^
Female	51 (43%)	23 (40%)	4 (11%)	
Male	69 (58%)	35 (60%)	33 (89%)	
Age (years)	28.7 ± 10.1	27.6 ± 10.0	25.1 ± 9.3	0.086
Age groups (years)				
<30	71 (59%)	42 (72%)	30 (81%)	0.026^c^
>30	49 (41%)	16 (28%)	7 (19%)	
Height (cm)	173.7 ± 9.2 (172.1–175.4)	175.2 ± 8.7 (172.9–177.4)	177.8 ± 8.8 (174.9–180.7)	0.052
Weight (kg)	74.4 ± 15.4 (71.6–77.2)	74.6 ± 13.6 (71.1–78.2)	79.8 ± 16.5 (74.3–85.3)	0.233
BMI (kg/m^2^)	24.6 ± 4.7 (23.8–25.5)	24.4 ± 5.0 (23.1–25.7)	25.1 ± 4.0 (23.8–26.4)	0.480
BMI groups (kg/m^2^)				0.590
<24.9	76 (63%)	42 (72%)	21 (57%)	
25–29.9	34 (28%)	13 (22%)	13 (35%)	
>30	10 (8%)	3 (5%)	3 (8%)	
Type of injury				0.728
High-impact sports	81 (68%)	36 (62%)	28 (76%)	
Low-impact sports	12 (10%)	6 (10%)	3 (8%)	
Not sports related	27 (23%)	16 (28%)	6 (16%)	
Mechanism of injury				**<**0.001^d^
Non-contact	109 (91%)	43 (74%)	17 (46%)	
Contact	11 (9%)	15 (26%)	20 (54%)	

^a^Continuous variables are shown as mean ± standard deviation (95% confidence interval), categorical variables are shown as number of patients and percentages per group.

^b^ *Post hoc* analyses revealed a significant difference between ‘no tear’ and ‘major tear’ (*P* < 0.001) and between ‘minor tear’ and ‘major tear’ (*P* = 0.002).

^c^ *Post hoc* analysis revealed a significant difference between ‘no tear’ and ‘major tear’ (*P* = 0.015).

^d^ *Post hoc* analyses revealed a significant difference between ‘no tear’ and ‘minor tear’ (*P* = 0.006), between ‘no tear’ and ‘major tear’ (*P* < 0.001), and between ‘minor tear’ and ‘major tear’ (*P* = 0.009).

**Table 3:** **Multivariate logistic regression** ^a^

**Variable**	**Group**					
	**‘Minor tear’**			**‘Major tear’**		
	**OR**	**95% CI**	***P*** **value**	**OR**	**95% CI**	***P*** **value**
Gender						
Female	Referent			Referent		
Male	1.37	0.51–3.67	0.530	7.38	1.97–27.61	0.003^b^
Age (years)	0.94	0.89–1.00	0.057	0.96	0.87–1.06	0.405
Age groups (years)						
<30	1.812	0.92–3.58	0.098	5.85	1.71–19.94	0.005^b^
>30	Referent			Referent		
Height (cm)	1.00	0.96–1.03	0.701	1.18	0.85–1.66	0.327
Weight (kg)	1.08	0.86–1.35	0.534	0.98	0.91–1.05	0.483
BMI (kg/m^2^)	0.95	0.83–1.08	0.438	1.02	0.91–1.15	0.735
BMI groups (kg/m^2^)						
<24.9	Referent			Referent		
25–29.9	0.32	0.07–1.42	0.134	1.49	0.03–4.38	0.837
>30	0.46	0.09–2.25	0.336	1.41	0.07–2.83	0.824
Type of injury						
High-impact sports	1.49	0.63–3.48	0.362	0.61	0.16–2.31	0.466
Low-impact sports	1.12	0.30–4.18	0.865	0.57	0.08–4.00	0.572
Not sports related	Referent			Referent		
Mechanism of injury						
Non-contact	Referent			Referent		
Contact	4.28	1.74–10.56	0.002^b^	18.49	5.96–57.37	**<**0.001^b^

^a^The reference group was ‘no tear.’

^b^Statistically significant.

## Discussion

The purpose of this study was to identify risk factors for different tear patterns of the lateral meniscus in ACL-injured subjects who underwent surgery within 6 months after injury. Identified risk factors for major meniscal tears were male gender with an OR of 7.38 and age of <30 years with an OR of 5.85. The strongest predictor for a major meniscal tear, however, was a contact injury mechanism with an OR of 18.49. The sole risk factor for a minor meniscal tear was a contact injury mechanism with an OR of 4.28.

Several other studies have evaluated risk factors for meniscus tears in ACL injuries before [27,28,25,29,30,4,31-33,16,34-39,6,40,17]. Most of these studies, however, focused on the association between the timing of surgery and meniscal tears. It has been clearly demonstrated that the incidence of medial meniscus tears increases with delayed surgery whereas the incidence of lateral meniscus tears is independent of the time interval from injury to ACL reconstruction [27,33,16,32,34,31,35,36,38,6,40,17]. This finding implies that lateral meniscus tears typically emerge during the initial injury and other factors than surgical delay must be responsible for lateral meniscus tears in ACL-injured subjects. However, only few studies have analyzed the association between different demographic and historical factors and meniscal tears [36,25,39]. A recent comprehensive study examined the predictors of meniscal tears in 541 patients undergoing ACL reconstruction [25]. The analyzed predictors were similar to our study and included age, sex, BMI, mechanism of injury, type of injury, interval from injury to surgery, and instability episodes. The authors found that male sex predicted more lateral and more medial meniscus tears, sports-related injuries predicted fewer medial meniscal tears, and more instability episodes predicted more medial meniscus tears [25]. In a similar study, the association between meniscal injuries accompanying ACL tears and the mechanism of injury, time from injury, activity level after the initial trauma, re-injury after the initial trauma, and BMI was analyzed in 293 patients [39]. The authors found increasing time from injury, active daily life, and re-injury to be risk factors for meniscal injuries [39]. A limitation of both studies is that meniscus tears were considered a binary finding (meniscus tear vs. no meniscus tear) and no differentiation was made between different patterns of meniscus tears. However, the pattern of meniscus tears observed in ACL-injured subjects varies greatly and a differentiated perspective seems to be necessary because of their potential prognostic value [41-43,14,5]. Our study is novel in analyzing the association of different tear patterns of the lateral meniscus and patient specific risk factors. Meniscus tear patterns regarded as major within this study were root tears, radial split tears, and unstable longitudinal tears including bucket-handle tears. These tear patterns have shown to dramatically alter the loading profile of the knee joint in biomechanical studies and are thought to promote the onset and rapid progression of OA [22,21,23,24,44]. Repair of major meniscal tears at the time of ACL reconstruction should therefore be preferred over meniscectomy, since meniscus repair is associated with less cartilage degeneration and better clinical results [12,45,7,46]. All patients within this study with major meniscal tears underwent meniscal repair. In a recent systematic review, the treatment of meniscus tears during ACL reconstruction over the last 10 years was determined [1]. Unfortunately, the authors found that meniscectomy was the most common method of treatment. Lateral meniscus tears were treated by meniscectomy in 71%, by repair in 14%, and left in situ in 14%. Interestingly, performing surgery within 6 weeks was predictive of more lateral meniscal repairs [1]. In addition, another study found higher meniscus healing rates in patients who underwent acute meniscus repair in conjunction with ACL reconstruction compared to delayed meniscus repair [47]. These findings underline the importance of early identification of patients with major meniscal tears if repair is attempted. The findings of the present study imply that male patients, patients under the age of 30, and particularly patients who sustained a contact injury are at high risk for an associated major lateral meniscus tear. We therefore recommend early referral of those patients to magnetic resonance imaging (MRI) and/or arthroscopy in order to allow meniscus repair in a timely manner.

A better understanding about factors associated with different patterns of lateral meniscus tears in ACL-injured subjects may also improve the diagnostic accuracy of MRI. Several studies have reported that the sensitivity and negative predictive value of MRI for meniscal tears decreases in the presence of ACL tears, particularly for the lateral meniscus [48-50]. A high index of suspicion is therefore necessary for adequate diagnosis. Based on the findings of the present study, special attention for lateral meniscus tears has to be given in males, patients <30 years, and patients presenting after a contact injury.

The present study found a contact injury mechanism to be the strongest risk factor for an associated major lateral meniscus tear. In contrast, other authors did not find that contact injuries predicted meniscus tears [25,39]. However, these studies did not differentiate between tear patterns and included both acute and chronic ACL injuries, which might be one explanation for the different results. In our opinion, the high risk for associated major lateral meniscus tears during contact injuries may be explained by higher forces applied to the knee joint compared to non-contact injuries. This assumption is supported by the observed higher incidence and severity of associated chondral lesions in contact ACL injuries [39]. With regard to the type of injury, we did not observe differences between high-impact sports-related, low-impact sports-related, and not sports-related injuries. This finding is in accordance with the results of other studies [25,31]. Other authors compared different injury patterns with regard to specific sports disciplines and reported that compared with soccer, skiing had an increased odds of isolated ACL injuries and other ligament injuries but decreased odds of meniscus and cartilage injuries, American football had a higher likelihood of having multiligament injuries compared with soccer, and basketball had a higher likelihood of having cartilage and lateral meniscus injuries than soccer [51]. Given the fact that a contact mechanism was the strongest risk factor for a major meniscal tear within the present study, we believe that the mechanism of injury might be more important than the type of injury.

Similar to our study, other studies have also found an association between male sex and an increased prevalence of concomitant meniscus tears [27,28,4,30,25]. However, these studies did not differentiate between different meniscus tear patterns. In the present study, male sex was only predictive for major meniscal tears whereas gender distribution was similar among patients with no tear or a minor tear. This association may be explained by a lesser degree of ACL resilience in females, leading to ACL rupture at smaller forces and thus less associated meniscal damage [28]. A higher failure load of the ACL may also explain the observed higher risk for associated major lateral meniscus tears in younger patients.

This study has several limitations that have to be considered when interpreting our results. First, this was a retrospective study and therefore the validity of our findings is limited. Second, the sample size of 215 patients is relatively low for an epidemiologic study. Third, only patients electing ACL reconstruction were evaluated and therefore the results may not allow generalization to all patients presenting with an ACL tear. Fourth, the injury mechanism was self-reported by the patients which might have introduced an information bias. Fifth, other factors not examined in this study may exist that predict the tear pattern, such as differences in knee morphology or material properties of the meniscus.

## Conclusions

Among patients with a tear of the ACL, male sex, age <30 years, and a contact injury mechanism are independent risk factors for concomitant major meniscal tears. Patients with a contact injury mechanism have an approximately 18-fold increased risk for a major lateral meniscus tear compared to patients with a non-contact injury. Special attention for this injury pattern is therefore necessary in those patients and early referral to magnetic resonance imaging and/or arthroscopy is recommended in order to allow meniscus repair in a timely manner.
